# Microstructural Change during the Interrupted Quenching of the AlZnMg(Cu) Alloy AA7050

**DOI:** 10.3390/ma13112554

**Published:** 2020-06-04

**Authors:** Thomas M. Kremmer, Phillip Dumitraschkewitz, Daniel Pöschmann, Thomas Ebner, Peter J. Uggowitzer, Gernot K. H. Kolb, Stefan Pogatscher

**Affiliations:** 1 Chair of Nonferrous Metallurgy, Montanuniversitaet Leoben, 8700 Leoben, Austria; phillip.dumitraschkewitz@unileoben.ac.at (P.D.); peter.uggowitzer@unileoben.ac.at (P.J.U.); 2 AMAG rolling GmbH, Postfach 32, 5282 Ranshofen, Austria; daniel.poeschmann@amag.at (D.P.); thomas.ebner@amag.at (T.E.); 3 Voestalpine Wire Rod Austria GmbH, Drahtstraße 1, 8792 St. Peter/Freienstein, Austria; gernot.kolb@voestalpine.com

**Keywords:** interrupted quenching, ageing, high strength Al alloys, transmission electron microscopy, atom probe tomography, fracture toughness

## Abstract

This study reports on the effect of interrupted quenching on the microstructure and mechanical properties of plates made of the AlZnMg(Cu) alloy AA7050. Rapid cooling from the solution heat treatment temperature is interrupted at temperatures between 100 and 200 °C and continued with a very slow further cooling to room temperature. The final material’s condition is achieved without or with subsequent artificial ageing. The results show that an improvement in the strength–toughness trade-off can be obtained by using this method. Interrupted quenching at 125 °C with peak artificial ageing leads to a yield strength increase of 27 MPa (538 MPa to 565 MPa) compared to the reference material at the same fracture toughness level. A further special case is the complete omission of an artificial ageing treatment with interrupted quenching at 200 °C. This heat treatment exhibits an 20% increase in fracture toughness (35 to 42 MPa m^−1/2^) while retaining a sufficient yield strength of 512 MPa for industrial applications. A detailed characterization of the relevant microstructural parameters like present phases, phase distribution and precipitate-free zones is performed using transmission electron microscopy and atom probe tomography.

## 1. Introduction

AlZnMg(Cu) alloys belong to the group of age-hardenable alloys and are characterized by high specific strength, sufficient ductility, and satisfactory corrosion resistance. Due to this combination of properties, they are widely used as rolled, pressed and forged products in structural applications, especially in aircraft constructions [1].

Recent developments focus on improving various performance criteria, particularly higher yield strength in combination with increased fracture toughness. These properties are highly dependent on the actual microstructure, which can be deliberately influenced by heat treatments. In particular, the controlled design of precipitate patterns during ageing procedures is of crucial importance. The precipitation sequence upon ageing is generally given by [2]:

SSSS → cluster → GP-zones (GPI, GPII) → η’ → stable η (MgZn_2_) or T ((Al,Zn)_49_ Mg_32_)

After solution heat treatment and quenching, the supersaturated solid solution (SSSS) develops cluster and Guinier Preston (GP)-zones during natural aging and in the early phase of artificial aging, respectively, which transform into metastable η’. This metastable phase precipitates in fine laths and is responsible for the main hardening effect, while the stable η or T phase can be observed at grain boundaries and during over-ageing. AlZnMg(Cu) alloys exhibit the highest strength in the peak hardened condition (T6) but can show stress corrosion cracking (SCC) in this temper. To avoid this detrimental effect the ageing time is extended (over-ageing, T7X). As a result of the over-ageing treatment, the strength decreases, but, simultaneously, the resistance to stress corrosion cracking increases [3,4].

For the design of precipitate patterns, a number of different strategies have proven to be effective. The following multiple-stage ageing treatments are of particular interest: RRA (Retrogression and Re-Ageing) [5,6,7,8] and IA (Interrupted Ageing) [9,10,11,12].

In RRA, a two-stage thermal cycle is applied to the alloy in underaged or T6 state. In the first stage, the retrogression or reversion, a high temperature is used; in the second stage, the usual T6 annealing treatment is applied again (re-ageing). In the first, higher temperature stage, the T6 precipitates dissolve, which is associated with a decrease in strength. In the second stage, the precipitates re-form and re-increase the mechanical strength. In this way, RRA preserves the T6 structure inside the grains. The precipitates at the grain boundaries, however, coarsen and resemble those of the T7 state, resulting in the desired effect of reduced SCC susceptibility [13].

In IA, which is a three-step treatment applied after solution annealing and quenching, the first ageing stage is carried out at higher temperatures and aims at the creation of GP-zones as a preliminary stage for the subsequent formation of precipitates. The second stage, at lower temperatures, is aimed at increasing the density of the precipitates and reducing the width of the precipitation-free zone (PFZ). The third step, again at a higher temperature, leads finally to the formation of a bimodal size distribution of the precipitates, thereby improving the tensile properties and the fracture toughness [9].

Like RRA and IA, the process of interrupted quenching (IQ) is a multiple-stage ageing procedure, in which the quenching process, starting from solution annealing temperature, is interrupted at a given IQ temperature, and is then continued to room temperature (RT) at a much lower cooling rate. Thereby or combined with optional subsequent natural and artificial ageing, a microstructure is to be created, which allows for a good combination of strength and toughness to be achieved. Studies on 6xxx alloys have already proved an increased age-hardening response when IQ was applied [14]. However, also, an improvement in the mechanical properties of AlZnMg(Cu) alloys using the example of AA7050 has already been reported [15].

The present study briefly illustrates the possible improvements in strength and fracture toughness through IQ treatment. A detailed examination of the corresponding microstructure is performed by transmission electron microscopy (TEM) and atom probe tomography (APT).

## 2. Materials and Methods

**Figure 1:**
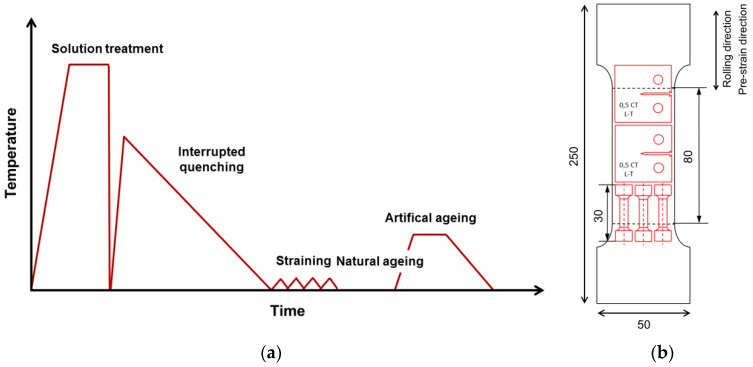
Schematic of the heat treatment during interrupted quenching (IQ) (**a**) [15] and sample geometries for mechanical testing specimens (**b**).

AA7050 alloy plates with the chemical composition given in Table 1 were provided by AMAG rolling GmbH, Austria. Figure 1 schematically illustrates the IQ processing steps. From the plate material, small bars were machined, with dimensions of 15 × 50 × 250 mm^3^. The samples were subjected to solution heat treatment at 482 °C in a circulating air furnace (Nabertherm N15/65 SHA, Lilienthal, Germany), followed by quenching in water at RT, with immediate reheating in two 40-mm-thick metal slabs [15]. Note that this was necessary because laboratory-scale quenching directly to the IQ temperature (e.g., in an oil bath) alters the quenching rate with changing IQ temperature. The cooling of the samples from IQ temperature (100 °C, 125 °C and 200 °C) to RT takes place freely in the center of a plate of 40 mm thickness, with a low cooling rate (described in [15]). Typically, the cooling time from the IQ temperature of 200 to 50 °C is about 5 h and roughly 3.5 h from 100 to 50 °C. The samples were further pre-strained to 1.5 % plastic deformation. Prior to the final artificial ageing (a.a.) at 125 °C for 24 h, natural ageing was applied for one week.

Mechanical properties were evaluated using static tensile testing according to ÖNORM EN ISO 6892-1:2017 20 01 [16] and fracture toughness test according to ASTM E399 [17]. For this purpose, samples were cut from the bar according to the schematic shown in Figure 1b.

The microstructural investigations included TEM and APT. Sample preparation for the TEM examination was done by punching 3-mm discs from the bulk alloy, and mechanical polishing with SiC paper up to a grit of 1200. Subsequent twin jet polishing with a Struers TenuPol 5 with a voltage of 15 V and at a temperature of −22 °C was carried out until an electron transparent area was obtained. The used electrolyte is common for the preparation of aluminium (66% methanol, 33% HNO_3_). On the samples scanning transmission electron microscopy high-angle annular darkfield (STEM-HAADF), electron diffraction and TEM brightfield investigations were performed using an FEI Tecnai F20 G2 (ThermoFisher, Hillsboro, OR, USA) with an acceleration voltage of 200 kV.

For the atom probe tomography (APT) measurements, small rods with a cross-section 1 × 1 mm^2^ were cut from the alloy in three different conditions: conventional T6, interrupted quenched to 125 °C (IQ125) and interrupted quenched to 125 °C plus artificial T6 ageing (IQ125 T6). These specimens were further thinned to a small tip by electropolishing with a 25% HNO_3_ in methanol electrolyte and in a second step with 2% perchloric acid in butoxyethanol [18]. The measurements were conducted on a LEAP ^TM^ 3000X HR atom probe (CAMECA Instruments, Madison, WI, USA) and the following experimental parameters were used: temperature (30 K), pulse frequency (200 kHz), pulse fraction (0.2), detection rate of 1% under ultra-high vacuum (10^−10^ mbar). The reconstruction and data analysis were carried out with the software package IVAS 3.6.12^TM^ from Imago Scientific Instruments Corporation (Madison, WI, USA) and additional custom scripts [19,20]. An optimization of the reconstruction parameters (field factor-k_f_, image compression factor-ICF) was done using spatial distribution maps (SDMs) [21].

## 3. Results

The following sections present the results of the tensile and fracture toughness tests, as well as the results of the structural characterization by scanning transmission electron microscopy and atom probe tomography.

### 3.1. Mechanical Properties

**Figure 2:**
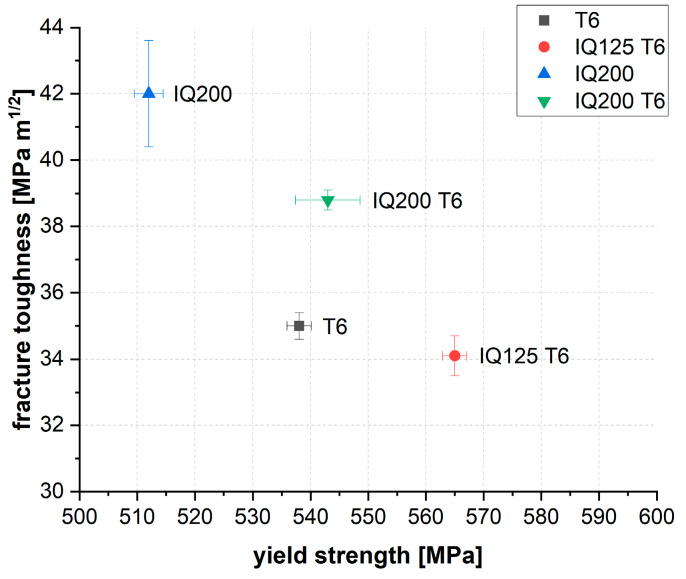
Mechanical properties of the alloy in different heat treatment conditions.

Figure 2 and Table 2 provide an overview of the mechanical properties achieved. The states T6 and IQ125 T6 exhibit comparable fracture toughness (34–35 MPa m^1/2^) but show a clear difference in the strength level. The yield strength of the IQ samples exceeds the T6 reference value by 27 MPa for IQ125 T6. The IQ200 and IQ200 T6 states, on the other hand, exhibit a noticeable increase in fracture toughness. For IQ200, the fracture toughness is, with 42 MPa m^1/2^, significantly above the T6 reference (increase by 20%) but is coupled with a slightly reduced yield strength (decrease by 5%). IQ200 T6, on the other hand, shows a marked improvement in toughness compared to T6, while the yield strength is comparable to that of the T6 state. Obviously, the additional thermal treatment (125 °C /24 h) applied to IQ200 leads to an increase in yield strength but simultaneously to a slight loss in toughness.

### 3.2. Transmission Electron Microscopy (TEM)

**Figure 3:**
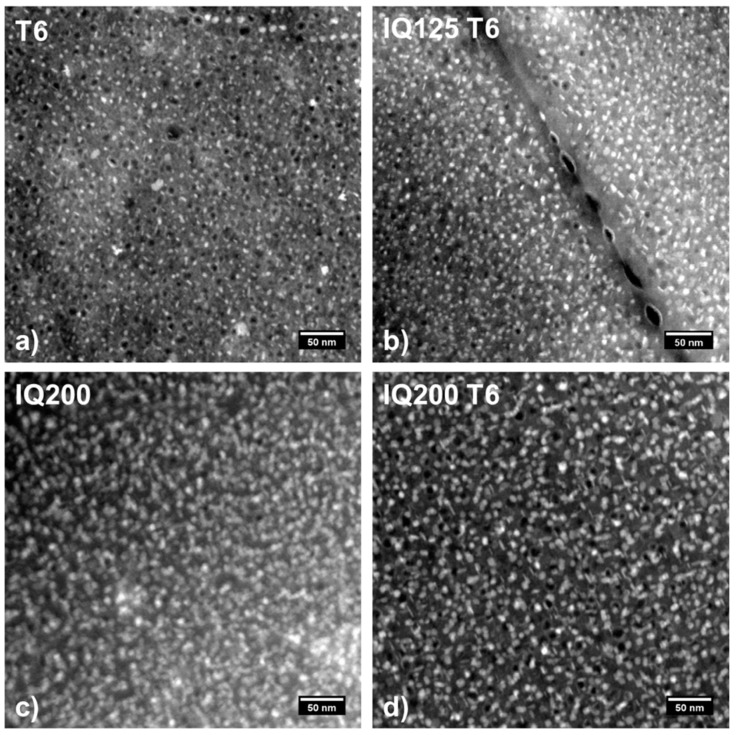
(**a**–**d**): Scanning transmission electron microscopy high-angle annular darkfield (STEM HAADF) images of hardening phases in the alloy AA7050 in a conventional T6 state (**a**) and after IQ125 T6 (**b**), IQ200 (**c**) and IQ200 T6 (**d**).

Figure 3a–d gives an overview of the shape and size of the hardening phases in the most interesting states T6, IQ125 T6, IQ200 and IQ200 T6. In the STEM HAADF images, the hardening phases are visible as small bright particles indicating heavy elements. Some images also display dark spots, which are caused by particles removed upon electropolishing.

The difference in the microstructure caused by the different heat treatments is clearly visible. Both the images of the T6 reference and the IQ125 T6 sample display a small and comparable particle size. The hardening phases in the material IQ200 without subsequent T6 treatment are larger compared to sole T6 and have a lower number density. No significant differences in size and number density of the hardening phases can be perceived for IQ200 and IQ200 T6. The increased particle size indicates that the IQ200 treatment leads to a more mature state compared to the T6 reference. This point will be discussed in more detail later.

**Figure 4:**
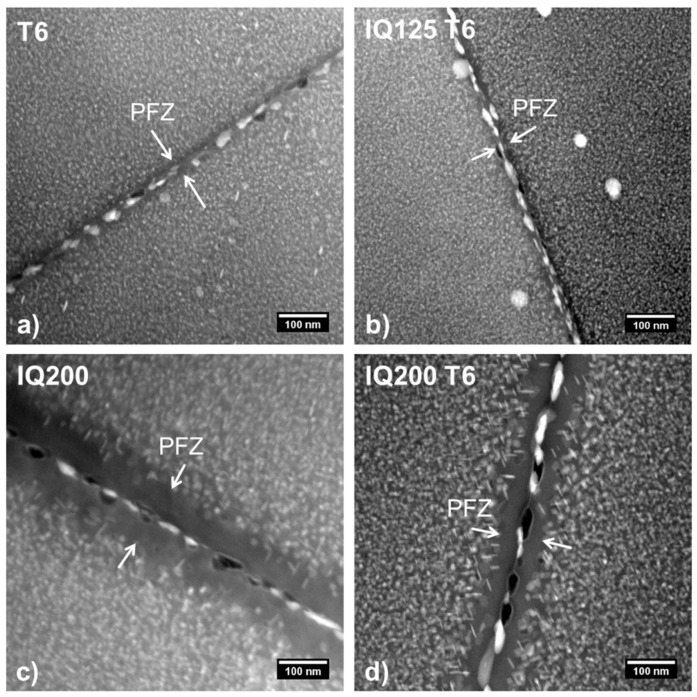
(**a**–**d**): STEM HAADF images of edge-on grain boundaries indication the precipitation-free zones (PFZs) in the alloy AA7050 in a conventional T6 state (**a**) and after IQ125 T6 (**b**), IQ200 (**c**) and IQ200 T6 (**d**).

The samples also greatly differ in the appearance of their grain boundary morphology. The differences are clearly visible in the overview shown in Figure 4a–d. Both the reference T6 and the IQ125 T6 have a small precipitation-free zone (PFZ) of comparable size along the grain boundaries. Contrastingly, the PFZs in the states IQ200 and IQ200 T6 are much more pronounced. However, the IQ200 T6 sample displays a less wide PFZ. Table 3 provides an overview of the exact widths of the precipitation-free zones, d_PFZ_. The measurement of the PFZ width was conducted on grain boundaries in edge-on configuration so the true width without geometrical distortion could be obtained. For each heat treatment, a minimum of eight grain boundaries with three measurements each at different positions were used for the statistical analysis.

**Figure 5:**
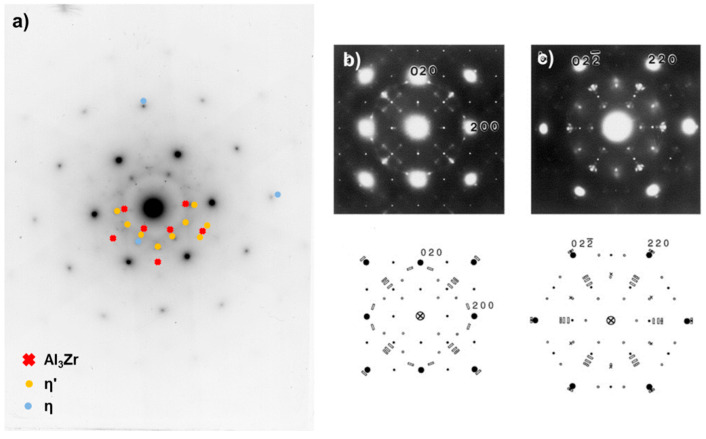
Diffraction analysis (selected area diffraction pattern) of the particles in sample IQ200 T6 and a comparison with literature data ([111]_Al_ projection). The results (**a**) and schematic representation from literature [24]: small open circles-η’, crosses–GP(II), rectangles–several orientation variants of η-MgZn_2_ (**b**,**c**).

To characterize the ‘mature state’ of the IQ200 T6 samples more precisely, a TEM diffraction analysis was performed. Figure 5 shows the results of the diffraction analysis of the IQ200 T6 state. The matrix reflexes are not marked and correspond to the {110} planes in the material. The used zone axis [B]-direction is [111]. The η’ metastable phase, η equilibrium phase and Al_3_Zr dispersoid are visible in this diffraction direction. For comparison, diffraction patterns in the [100] and [110] direction were also obtained. These three low-index zones are well quantified by literature and simulations and are often used for this type of analysis [22,23,24,25]. The results clearly show that, in the interrupted quenched IQ200 T6, both metastable η’ and stable η are present. It can be assumed that the stable phase is preferably formed at the grain boundary. The ageing process here is obviously already quite advanced and resembles an overaged T7 state [26] but also an RRA state [27]. From the size of the phases displayed in Figure 4c,d, it might be concluded that, even in the IQ200 state, both phases, metastable η’ and stable η are present.

### 3.3. Atom Probe Tomography (APT)

From Figure 1 and Table 2, it can be seen that the states T6, IQ125 and IQ125 T6 differ in their yield strength, but their microstructures revealed by TEM (Figure 4a,b) do not allow for sufficient differentiation. The question now arises as to whether interrupted quenching results in significant particle formation at all, or whether these are only formed during the subsequent T6 treatment. Therefore, we conducted an APT study with the states ‘reference T6’ and interrupted quenching to 125 °C, once without T6 treatment (IQ125) and once with additional T6 treatment (IQ125 T6).

**Figure 6:**
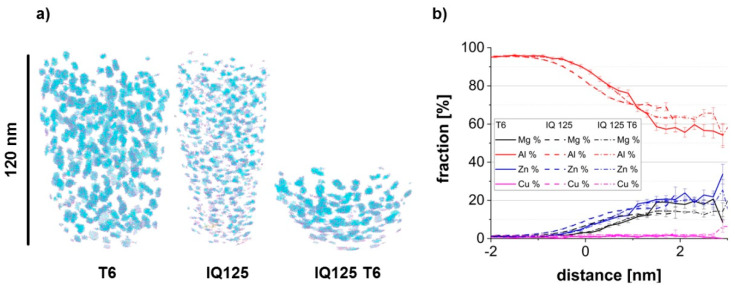
(**a**) Three-dimensional (3D) reconstructions of the atom positions for identified precipitates via the cluster search. (**b**) Proximity histogram deduced from Mg-Zn iso-surfaces at 10 at% for Al (red), Zn (blue), Mg (black) and Cu (purple).

Figure 6a shows 3D reconstructions of the atom positions of identified precipitates of the three different materials conditions. In Figure 6b, a proximity histogram of such particles is presented. Precipitates are present in all tested states, i.e., also in the IQ125 sample, which means that the thermal load during the slow cooling from the IQ temperature was sufficient to form a hardening phase. However, a clear difference can be seen between IQ125 and both T6 conditions. The precipitates in the IQ125 sample are much smaller (Figure 6a) and less enriched in solutes, as visible in the proximity diagram (Figure 6b).

**Figure 7:**
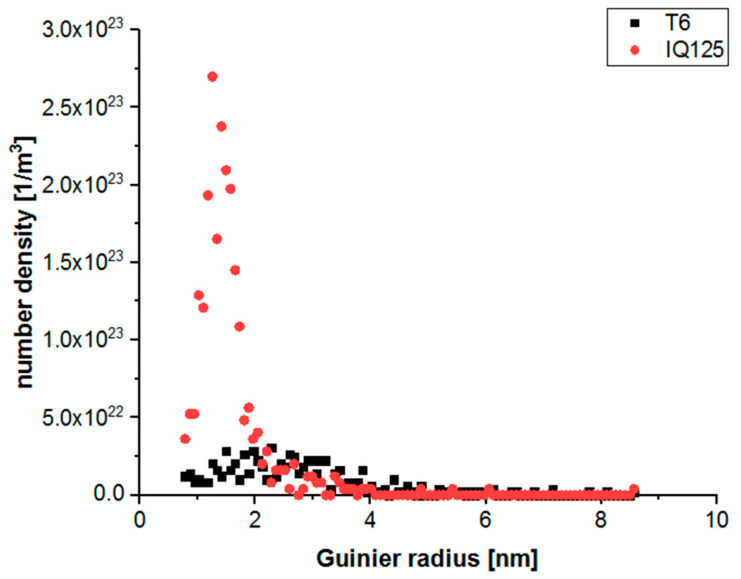
Size distribution of the precipitates in T6 and IQ125, expressed as Guinier radius. T6 state in black and IQ125 in red.

The precipitates were identified within the measured data set with a number density of 7.5 × 10^23^ (1/m^3^) for the T6 state, 2.3 × 10^24^ (1/m^3^) for the IQ125 state and 1.24 × 10^24^ (1/m^3^) for the IQ125 T6 state. Here, the cluster search algorithm of [20] was applied with Zn, Mg and Cu atoms as possible core atoms. The average Guinier radii [18] were calculated, and their distribution for T6 and IQ125 is shown in Figure 7. Obviously, the state IQ125 is characterized by a bimodal distribution, with a few larger and many very small precipitates. With such size distribution, IQ125, as a preliminary stage to IQ125 T6, can thus be compared with interrupted ageing (IA) after the second low-temperature annealing stage [9].

From the APT data, the average composition of the precipitates was also evaluated via the built-in functions “alphaShape” and “inHull” of MATLAB^TM^ [28]. The Zn and Mg content is with ≈ 20 at% and ≈ 15 at%, respectively, approximately the same in both states T6 and IQ125 T6.

## 4. Discussion

Investigations into the microstructural characteristics revealed the following picture: (i) interrupted quenching to low temperatures (in the region of the usual artificial aging temperature of typically 125 °C) leads, after subsequent T6 annealing, to a higher yield strength compared to conventional T6 treatment, but fracture toughness remains at a comparable level; (ii) interrupted quenching to high temperatures (≈200 °C) results in remarkable increase in fracture toughness, with simultaneously minor reduction in yield strength if a subsequent T6 treatment is waived, or even a slight increase in yield strength if a subsequent T6 treatment is applied.

The magnitude of hardening as a function of precipitate size, d [m], and their number density, N_v_ (1/m^3^), can be estimated after [29,30] with:

(1) $$\Delta R_{p}\propto \alpha M\mu b{\left(N_{v}d\right)}^{0.5}$$

where α is the obstacle strength of the precipitates, M is the Taylor factor (=3.1), μ is the shear modulus (=27 GPa) and b is the length of the Burgers vector (=0.286 nm). For T6 hardening to 540 MPa, an assumed matrix strength of 150 MPa yields an increase in the strength of $\Delta R_{p}=$ 390 MPa. According to Equation (1), such an increase can be calculated with an obstacle strength of 0.3. If it is further assumed that the precipitates in the IQ125 T6 state have an obstacle effect comparable to that of T6, their increased number density results in an increase in strength of 440 MPa, which corresponds fairly well with the experimental data. The lower yield strength in the IQ200 and IQ200 T6 states, can be easily explained by the lower number density of precipitates compared to T6 and IQ125 T6 (Figure 3).

When selecting AA7050 plates, not only the strength but, in particular, the fracture toughness is of decisive importance. This is where the superiority of the IQ200 states becomes apparent. The gain in toughness compared to the higher strength conditions is significant and amounts to 7 MPa m^1/2^ (+20%) for IQ200 and ≈3.8 MPa m^1/2^ (+10%) for IQ200 T6. Hornbogen and Starke [31] as well as Dixit et al. [32] have discussed and evaluated the fracture mechanism that is dominant in the presence of PFZ. Here, the fracture toughness is controlled by void nucleation and results to:

(2) $$K_{\mathrm{IC}}\propto {\left(\frac{R_{p}{\mathrm{Ad}}_{\mathrm{PFZ}}}{D}\right)}^{0.5}$$

where R_p_ (MPa) is the yield strength, A the elongation to fracture (−), d_PFZ_ (m) the width of PFZ and D (m) the average grain size. Since the grain size remains unchanged when different hardening strategies are applied, the values given in Table 2 and Table 3 can be used to create a ranking according to the value of the fracture toughness: IQ200 is clearly ahead of IQ200 T6, followed at a considerable gap by equal values of T6 and IQ125 T6. The advantage of IQ200 over IQ200 T6 is clearly due to the higher value of d_PFZ_. With the added T6 treatment, the width of the PFZ is reduced. After IQ to 200 °C and the subsequent slow cooling, there are still enough atoms in solid solution, so that additional precipitation formation takes place during the T6 annealing treatment, leading not only to an increase in particle number density but also to a reduction in d_PFC_. The result is an increase in strength with a simultaneous slight reduction in fracture toughness.

The results illustrate that the application of interrupted quenching offers an interesting heat treatment option, which allows for targeted improvements of various material properties. Just by IQ treatments at 200 °C, without subsequent artificial ageing, a stable microstructure is produced, which combines sufficient strength with good fracture toughness. The omission of the T6 annealing step (24 h at 125 °C) has huge effects in terms of cost savings during production. Also particularly noteworthy is the formation of a microstructure similar to that of over-ageing or RRA. Thus, it is not at all surprising that these processes also have a comparable influence on the strength–toughness behavior [33,34]. The similar microstructure achieved with IQ at 200 °C compared to those processes also gives reason to anticipate an interesting stress corrosion cracking response, which should be investigated in future studies.

## 5. Conclusions

The use of Interrupted Quenching provides an attractive compromise between strength properties and fracture toughness in the production of 7xxx high-strength plate alloys. As the yield strength of the material is influenced by the distribution of the hardening phases while the fracture toughness is governed by the void nucleation (see Discussion Section), it is theoretically possible to improve the fracture toughness without significant loss in strength and, vice versa, increase the strength level without loss in toughness. Interrupted Quenching leads to similar microstructures and properties as obtained by Interrupted Ageing at low temperatures and over-ageing or Retrogression and Re-ageing at higher temperatures. However, the process management of IQ is much more attractive than the complex and long-lasting procedures IA and RRA.

With an optimized treatment at 125 °C and subsequent artificial ageing, it is possible to obtain a strength increase of 27 MPa (538 MPa to 565 MPa) compared to a standard T6 treatment without loss in fracture toughness. This increase in strength can be correlated with the slight precipitation density increase from 7.5 × 10^23^ (1/m^3^) for T6 to 1.24 × 10^24^ (1/m^3^) for IQ125 T6. The higher density may be caused by the slow cooling from 125 °C to room temperature during interrupted quenching before the artificial ageing process, which is done at a constant temperature. For both material states (T6 and IQ125 T6), there is no significant change in the PFZ width. Contrary to this, the IQ process at 200 °C causes an increased PFZ for both states with and without subsequent artificial ageing. This is especially visible in the IQ200 sample, exhibiting a PFZ of approximately 100 nm (compare Table 3), which is three times as wide as the reference T6 state. The high PFZ width comparable to an over-aged state improves the fracture toughness resistance by 20% (from 35 to 42 MPa m^1/2^). The fact that the interrupted quenching is conducted at an elevated temperature (200 °C) compared to the peak artificial ageing temperature (125 °C) causes precipitate growth and finally decreases the yield strength to 512 MPa. Particularly noteworthy, this strength level is still sufficient for most applications and the complete omission of artificial ageing offers an attractive economic advantage.

## Figures and Tables

**Table 1:** Overall chemical composition (wt%, average of 4 measurements) of the material investigated.

Material Designation	Zn	Mg	Cu	Zr	Al
AA7050	6.44 ± 0.04	2.04 ± 0.02	2.50 ± 0.02	0.11 ± 0.001	Balance

**Table 2:** Mechanical properties in the long transverse (LT) direction of the T6 reference and IQ-treated samples.

Sample	R_p_ (MPa)	R_m_ (MPa)	A (%)	K_Ic_ (MPa m^−1/2^)
T6	538	581	17.8	35.0
IQ125 T6	565	606	17.5	34.1
IQ200	512	563	17.1	42.0
IQ200 T6	543	591	13.7	38.8

**Table 3:** Width of the precipitation-free zone, d_PFZ_ (nm).

T6	IQ125 T6	IQ200	IQ200 T6
31 ± 9	28 ± 4	117 ± 15	96 ± 13
